# Assessing osseointegration of metallic implants with boronized surface treatment

**DOI:** 10.4317/medoral.23175

**Published:** 2020-04-09

**Authors:** Lukasz Witek, Nick Tovar, Christopher D. Lopez, Jonathan Morcos, Michelle Bowers, Roumiana S. Petrova, Paulo G. Coelho

**Affiliations:** 1 Department of Biomaterials and Biomimetics, New York University College of Dentistry, New York, NY; 2 Department of Plastic and Reconstructive Surgery, Johns Hopkins School of Medicine Baltimore, MD; 3 Department of Chemistry and Environmental Science, New Jersey Institute of Technology, University Heights Newark, NJ; 4 Hansjörg Wyss Department of Plastic Surgery, New York University School of Medicine, New York, NY; 5 Department of Mechanical Engineering, Tandon School of Engineering, New York University, New York, NY

## Abstract

**Background:**

Modification of endosteal implants through surface treatments have been investigated to improve osseointegration. Boronization has demonstrated favorable mechanical properties, but limited studies have assessed translational, *in vivo* outcomes. This study investigated the effect of implant surface boronization on bone healing.

**Material and Methods:**

Two implant surface roughness profiles (acid etched, machined) in CP titanium (type II) alloy implants were boronized by solid-state diffusion until 10-15µm boron coating was achieved. The surface-treated implants were placed bilaterally into 5 adult sheep ilia for three and six weeks. Four implant groups were tested: boronized machined (BM), boronized acid-etched (BAA), control machined (CM), and control acid-etched (CAA). Osseointegration was quantified by calculating bone to implant contact (BIC) and bone area fraction occupancy (BAFO).

**Results:**

Both implant types treated with boronization had BIC values not statistically different from machined control implants at t=3 weeks, and significantly less than acid-etched control (*p*<0.02). BAFO values were not statistically different for all 3-week groups except machined control (significantly less at *p*<0.02). BAFO had a significant downward trend from 3 to 6 weeks in both boronized implant types (*p*<0.03) while both control implant types had significant increases in BIC and BAFO from 3 to 6 weeks.

**Conclusions:**

Non-decalcified histology depicted intramembranous-like healing/remodeling in bone for controls, but an absence of this dynamic process in bone for boronized implants. These findings are inconsistent with *in vitro* work describing bone regenerative properties of elemental Boron and suggests that effects of boron on *in vivo* bone healing warrant further investigation.

** Key words:**Boronization, acid-etched, machined, implants, osseointegration, in vivo, solid-state diffusion.

## Introduction

Successful endosteal implant placement is measured by a desired bone healing response that results in predicTable long-term implant stability and function. This necessary response, termed osseointegration, is governed by the tissue-implant surface interface, implant geometry, surgical procedure/placement and the bone remodeling processes over time (1). Bone remodeling, initiated post-surgically and affected by primary and secondary stability, leads to the adaptation and healing of bone at the interface of the implant. The biological interface, composed of cellular and extracellular structures, is directly exposed to the surface chemistry of the implant (2). The successful application of these principles has resulted in over 2.2 million pounds of titanium devices implanted into patients worldwide every year. However, implant fatigue continues to be an important cause of implant failure, with reports in the dental and orthopaedic literature alike (3,4), citing the need for manufacturing procedures that can strengthen and improve osseointegration and wear resistance.

Numerous studies have focused on techniques to improve osseointegration and wear, such as implant surface treatments. Surface modification can significantly alter its physico-chemical properties without altering desired bulk properties (5). This addresses the need for superior integration between bone and implant while retaining desired bulk characteristics of an implant material. Chemical and/or physical surface treatments such as hydroxyapatite coating, acid etching, and sandblasting have all shown various degrees of increased initial stability *in vivo* when applied to metallic implants (6). In living systems, where implant-bone remodeling has been shown to continue up to 5 years when stimulated by masticatory loading, the ability of energy absorption is an important factor in the long-term success of the implant (7). It is therefore important to improve the tribological, wear properties, and surface treatment methods of implants, in both dental and orthopaedic markets. Methods including laser, plasma surface treatments, ion nitriding and solid-state diffusion have all been investigated to achieve these goals.

Boronization, or boriding, is a thermo-chemical treatment in which boron atoms diffuse into metals resulting in a nanocrystalline hardening surface layer (8). The boron atoms are limited to the nanocrystalline layer and form a range of hard metal boride phases, which has been shown to contribute high wear resistance, corrosion resistance, and up to a ten time increase in service life of implants. Conventional processes available for boronizing vary but the most widely used is pack boronizing, which uses solid precursors in powder form to undergo solid-state diffusion. This process is inexpensive and simple/flexible in deployment, thereby allowing for the coating of complex geometric bulk constructs.

The translational applications of boronized titanium implants and bone healing have not been extensively explored *in vivo*, but *in vitro* studies have shown a favorable cell growth rate, with excellent blood compatibility with low hemolysis level (<0.12%) on plasma sintered composite TiB2-Ti within a 48-hour duration when compared with CP Ti (0.17%) and Ti-6Al-4V (0.36%). *In vivo* investigations testing the effect of boron incorporation to PLGA scaffolds for hard tissue healing proved promising as bone mineralization density and computed tomography analysis proved that scaffolds with boron increased healing rates of bone defects in a rat femur (9). Boron significantly increased levels of mineralization and bone associated protein expression in osteoblast formation, bone densification, mineral content, and mechanical properties. *In vivo* work has described beneficial effects of dietary boron on bone strength in rabbit models (10). Despite these promising systemic effects, the effects of boron on healing through localized biomaterial delivery have not been investigated in larger models to date.

The present study explored the effect of boriding on the early osseointegration of titanium implants in a highly translational large animal model. The hypotheses of the study were (i) that boron containing surfaces will present similar osseointegration degrees to titanium surfaces, and that (ii) acid etched surfaces will present higher osseointegration degrees relative to machined surface for both boron containing and non-boron containing groups.

## Material and Methods

- Boronization Treatment

Prior to boronization treatment, the Type II CP titanium implants (4mm diameter and 10mm length) in this study were first cleaned with acetone in an ultrasound bath, dried with compressed air, and then chemically etched for thirty seconds with a solution mixture of Nitric acid and Hydrofluoric acid mixed in a 10:1 ratio. Afterwards, the implants were washed in distilled water, soaked in acetone, and left to air dry.

Titanium implant boronization was performed by solid state diffusion using a powder mixture of 97% weight amorphous boron (SB Boron 90, SB Boron Corporation), 2% weight boron carbide (240 grit, technical grade, Electro Abrasive Corp.), and 1% weight potassium fluoroborate (KBF4) (Alfa Aesar) as an activator. The boron powder mixture was dried in a box furnace (ThermoLyne, model 48015) at 250°C for two hours. After the heating process was completed, the mixture was removed from heat to permit cooling.

A ceramic process crucible was filled halfway with the dried powder mixture; the titanium coupons were placed in the crucible approximately 1 cm apart, and fully submerged by approximately 10 mm from in the ceramic crucible. The remaining dried powder mixture was then placed on top of the titanium coupons to fill the crucible. Upon complete filling of the crucible, the crucible was shaken to make sure the powder completely surrounded the coupons and any residual voids in the powder solution.

The filled crucible was then placed into a vacuum furnace (CM Furnaces Corp.,) at 250°C for four hours. Next, the temperature was increased to 1100°C and the diffusion process was carried over 8 hours in an argon technical grade atmosphere with the flow of argon 5 m3/h. After eight hours, the furnace was cooled down to 250°C while still under an argon atmosphere, and then the furnace was turned off. After the furnace cooled down to room temperature, the boronized implants were removed from the powder mixture and cleaned with methanol in an ultrasound tank.

In order to measure the thickness of the boron coat achieved, cross sections were made, samples were etched, and a Zeiss Optical Microscope was used to measure the coat. The thickness of the boron coating was 10-15µm. After the treatment process and prior to any implantation were subjected to sterilization by gamma irradiation (CellRad Faxitron Tucson, AZ) at a dose of 25 kGY.

- Surgical Approach

An ovine hip model was used due to the low-density bone conFiguration and size, which allowed for placement of all experimental groups within each subject minimizing the number of animals used. The study was conducted in accordance with ethical guidelines from the Institutional Animal Care and Use Committee under ARRIVE guidelines (Comité d’éthique Anses/ENVA/UPEC Approval Reference#: 13-011). Following the approval from the committee a total of five male sheep (each weighing ~55kg) were acquired and allowed to acclimate for 7-days at the facility. After the acclimatization period the sheep were subjected to the previously approved protocol for the surgical procedures for the sheep ilia. Due to the size of the iliac crest in the species, experimental groups were nested within subject, allowing for an increase in statistical power and decrease in the number of animals. Four implants were inserted in sheep ilia bilaterally yielding to 40 implants total (n=20/per time *in vivo*) (20 implants with surface treatments and 20 control). Two time points were analyzed in this study, the first surgical procedure was performed on the left hip, which provided the 6-week time point, and subsequently 3-weeks after the initial surgical procedures the right hip was operated in order to provide the 3-week *in vivo* time point. The study comprised of four different groups of implants to be analyzed in this experiment: boronized machined (BM), boronized acid-etched (BAA), control machined (CM), and control acid-etched (CAA).

Prior to surgery, anesthesia was induced with sodium pentothal (15-20 mg/kg) in Normasol solution into the jugular vein and maintained with isofluorane (1.5-3%) in O2/N2O (50/50). Animal monitoring included ECG, end tidal CO2, and SpO2 and body temperature, which was regulated by a circulating hot water blanket. Prior to surgery, the surgical site was shaved and iodine solution was applied to prepare surgical site. A ~10 cm incision was made along the iliac crest, adipose and muscular tissue dissected, exposing the ilium (11). Drilling was performed at 1100 rpm under saline irrigation. The final diameter of drills utilized was 3.8 mm. Individual implant group position within the ilia were interpolated as a function of animal subject to minimize location bias. Layered closure was performed. Cefazolin (500 mg) was administered intravenously pre-operatively and post-operatively. Post-operatively, sheep were transported to their stall for recovery, with food and water provided *ad libitum*. At the designated time point the animals were euthanized by anesthesia overdose, which included the administration of a combination of Telazol (2–5 mg/kg; intramuscular) and Xylazine (2 mg/kg; intramuscular), followed by the placement of an intravenous catheter in the ear, used to administer 120 mg/kg, intravenously, of sodium pentobarbital. Death was confirmed by auscultation, absence of heartbeat, and EKG. The samples were retrieved *en bloc* and placed in formalin solution for 24 hours followed by ethanol solution until histological processing.

- Histological Preparation and Histomorphometry 

Each experimental group was processed for histologic evaluation via progressive dehydration and infiltration using ethanol and methyl salicylate, respectively, followed by final embedding in methyl methacrylate (MMA) as previously reported. The blocks were then cross-sectioned along the long axis of each implant with a slow-speed diamond saw (Isomet 2000, Buehler Ltd., Lake Bluff, IL, USA) to create slices as thin as 100 micrometers(μm). These histologic cuts were glued to an acrylic slide with acrylate-based adhesive (Loctite prism 408 industrial adhesive) and subsequently grinded and polished, with water irrigation, using progressively finer silicon carbide (SiC) abrasive papers (600, 800, and 1200) (Metaserv 3000, Buehler Ltd., Lake Bluff, IL, USA) to achieve a final thickness of ~75 μm. Final sections were stained with Stevenel’s Blue and Van Gieson’s *Pi*cro Fuschin (SVG) stains. Histological observations and images were digitalized using an automated Aperio slide scanning system and accompanying computer software (Aperio Technologies, Vista, CA, USA). Osseointegration and bone growth was quantified and evaluated using a specific image analysis software (ImageJ, NIH, Bethesda, MD). A single user blinded to the implant groups and distribution, quantified bone-implant contact (BIC) and bone area fraction occupancy (BAFO) to determine osseointegration level of implants. BIC quantifies the degree of osseointegration by calculating the percentage of bone in contact with the implant’s perimeter, while BAFO quantifies bone within the implant thread.

- Statistical Analysis

All histomorphometric and biomechanical testing data are presented as mean values with the corresponding 95% confidence interval values (mean ± 95% CI). %BIC and %BAFO data were analyzed using a linear mixed model with fixed factors of implant surface texture (AA and M), boronization (B) presence or absence, control (C) and time point (3 and 6 weeks). All analysis was completed with IBM SPSS (v23, IBM Corp., Armonk, NY).

Results 

**Figure 1:**
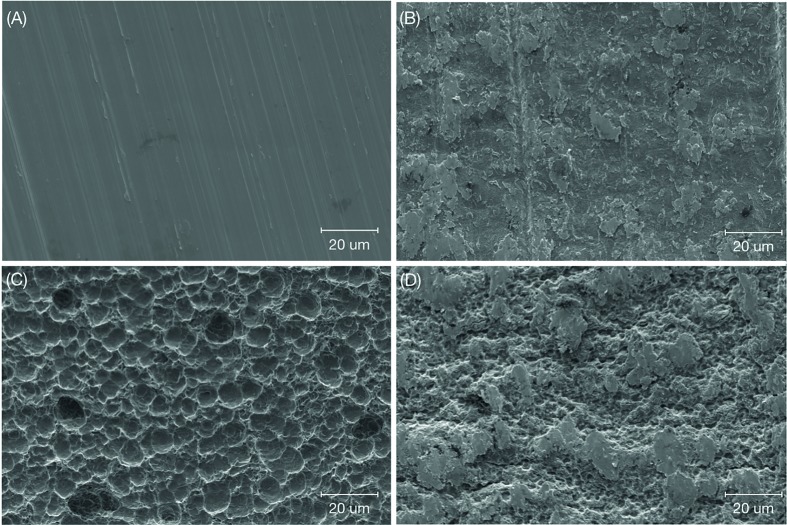
Scanning electron micrograph of: (A) machined titanium implant surface indicating the commonly seen grooved appearance (control machined, (CM)), (B) boronized machined (BM), (C) acid etched titanium implant surface showing the distribution of small peaks and divots (control acid-etched (CAA)), and (D) boronized acid-etched (BAA).

Qualitative surface characterization was accomplished using scanning electron microscopy (SEM) (Hitachi S3500N) and was performed at various magnifications under an acceleration voltage of 5kV to observe the surface topography of each group (n = 1 per surface) (Fig. 1. As seen in the SEM micrographs, the control acid-etched (CAA) (Fig. 1) illustrated a surface with a more pronounced topography in comparison to the smooth surface of the control machined (CM) implant with its machined grooves (Fig. 1).

No evident signs of inflammation or infection were observed during immediate and longer-term post-operative routines. After necropsy, clinical stability was detected for all implants.

**Figure 2:**
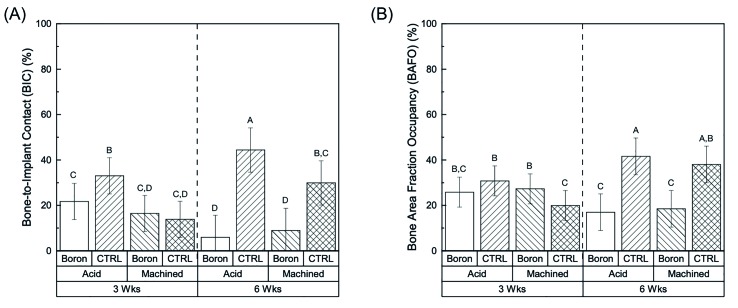
BIC (a) and BAFO (b) as a function of time points (three vs. six weeks) and surface treatment (acid etched vs. machined) within each group (boronization vs. control). Letters indicate statistically homogenous groups.

The histomorphometric results demonstrated no significant differences for bone to implant contact (BIC) and bone area fraction occupancy (BAFO) values for boronized implant groups as a function of surface texture (as machined (M) vs acid etched (AA)) at the individual time points, 3- and 6-weeks (*p*>0.05) (Fig. 2). At 3 weeks, only a significantly higher BIC value was observed for the CAA group (*p*<0.02) while the BAA, BM and CM values showed no significant differences (Fig. 2). On the other hand, when evaluating as a function of time (3- vs 6-weeks) significantly (*p*<0.01) higher values of BIC were observed for the BAA group between at the earlier time point (3-weeks), 21.73%±7.9 vs 5.93%±9.7 (6-weeks), while no statistical differences were observed for BM samples between the time points (16.44%±7.9 (3 weeks) vs 8.9%±9.7 (6 weeks)) (Fig. 2). When evaluating for all factors (time, surface texture, and presence or absence of boron on the surface) mean BIC values for the BM implants showed no significant differences, between 3 and 6-weeks, while BAA resulted in significant decrease from 3- to 6-weeks (Fig. 2).

Subsequently, when evaluating for BAFO of the respective implant groups, significantly (*p*<0.02) higher values were observed for BAA (25.82%±6.6), BM (27.29%±6.6) and CAA (30.76%±6.6) vs the CM (19.92%±6.6) group at 3 weeks (Fig. 2). When evaluating BAFO as a function of time *in vivo* (3- vs 6-weeks), a significant (*p*<0.03) decrease for BAFO was observed for both the BAA and BM groups (Fig. 2), while a significant increase was detected for both CAA and CM groups (Fig. 2).

**Figure 3:**
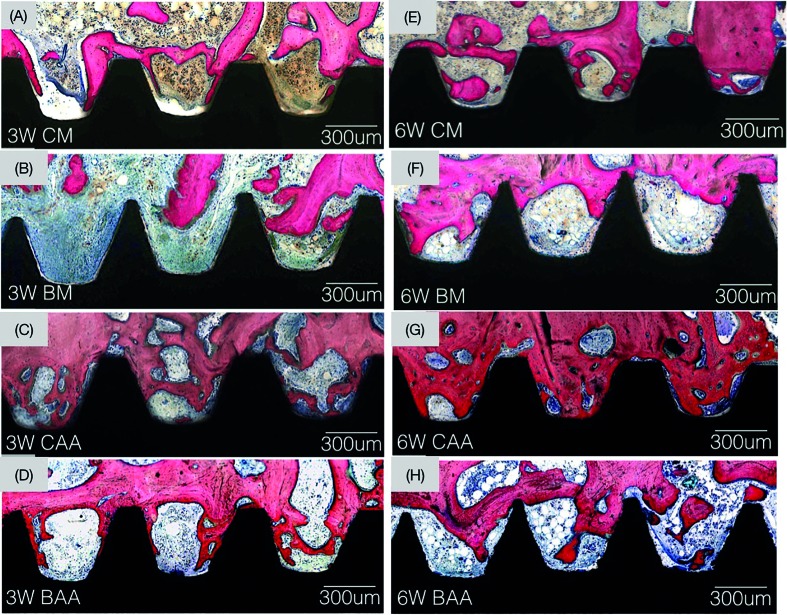
Optical micrographs taken at 3- and 6-week time point at healing chamber and bone interface. (A) Control-machined implant group. 3-weeks: (B) Boron-machined implant group. (C) Control acid-etched implant group. (D) Boron acid-etched implant group. 6-week: (E) Control-machined implant group, (F) Boron-machined implant group, (G) Control acid-etched implant group, and (H) Boron acid-etched implant group.

Histological observation at various magnifications supported the histomorphometric BIC and BAFO statistical findings. While new bone growth was observed in proximity and in contact with all groups investigated at 3 weeks (Fig. 3), the amount of bone contact and fraction occupancy between threads decreased for the boron diffused surfaces (Fig. 3). The amount of bone observed in contact for the control implants increased over time. From a morphological perspective, normal progressive remodeling of bone is observed for the control groups (Fig. 3), such dynamic change were not observed for the boron containing groups over time (Fig. 3).

## Discussion

The effects of boronization on metallic material properties are both well-documented and favorable. The aim of this study was to elucidate the effect of boronization on osseointegration. Boriding implant surfaces prior to surgery affected the osseointegration of the implants for at least six weeks *in vivo*, as supported by decreased BIC and BAFO when compared to control groups, as well as diminished overall osseointegration, and demineralization.

Surface treatment with boron produces a TiB phase on titanium substrates that can improve the surface hardness, mechanical properties, corrosion resistance as well as good cytocompatibility. Similar to nitriding and carburizing, boronizing is a thermo-chemical heat treatment used to improve the wear and corrosion resistance over decades of use. Indeed, boride coating has actually been shown provide greater surface hardness and higher wear resistance than nitride and carbide coatings. However, while findings are well-established in materials science investigations, translational applications have been more limited. In the present translational study, the application of these material science properties into a biological system resulted in unfavorable healing for up to six weeks *in vivo*, suggesting that factors that have previously not been considered when attempting to boride merit investigation, such as ideal concentration, and the effect of boron on indirect mediators of bone healing, such as osteoclast/osteoblast precursors and macrophages.

The limited number of small animal model studies investigating the role of boron for bone repair have showed that boron has osteogenic potential. Chen *et al*. used boron-coated mesoporous bioactive glass to improve the bone regenerative capacity and mechanical properties of bone derived from a novel nanogel (12). Gorustovic *et al*. implanted boron-modified bioactive glass particles in rat tibia bone marrow and determined that greater degrees of bone with higher calcium to phosphorus ratios formed consequent to boron (13). Other studies have demonstrated that tissue-engineered polymeric scaffolds infused with boron significantly increase osteoblast activity in rabbit calvaria, thereby warranting the investigation of these principles in larger, more translational animal models in this study. Although the treatment of devices with boron in low doses for bone tissue engineering has been previously demonstrated by Hakki *et al*., there currently exists no evidence that the incorporation of boron has exerted cellular or systemic toxicity.

Implants subjected to boronization unexpectedly declined in bone formation at the implant surface over time. At six weeks *in vivo*, there was significantly less bone growth within the implant healing chambers measured by BAFO analysis and observed histologically. Acid-etched implants subjected to boriding also exhibited this same trend to an even greater degree. This is likely attributed to the fact that acid-etching increases surface area of the implant, thereby subjecting bone to an even greater degree to boron diffusion. Of note, demineralized regions of woven bone growth were noticed in the BAE group focally within bone remodeling regions and not mature lamellar bone. This suggests that the effects observed may be linked to early stages of bone remodeling. These unexpected outcomes are inconsistent with previous reports highlighting the important role of boron in bone homeostasis (14) and warrant future investigation of ideal and supra-physiologic boron diffusing from the TiB2/TiB micron deep implant surface at local bone sites, as well as the potentially toxic effects on bone formation.

The study was effective in determining the effect that boronizing metal implants with various surfaces have on new bone growth and osseointegration post-surgically. The initial hypothesis of increased integration was disproven at later time points, as the boronization decreased new bone growth and even demineralized pockets of woven bone back to the osteoid state. This result could potentially be applicable in numerous pathologic states of bone overgrowth. Also, further boronization studies are needed to assess the ideal processing parameters that would allow for increased bone growth and tribological surface properties without negative effects on bone remodeling.
